# Cord Blood Levels of EPA, a Marker of Fish Intake, Correlate with Infants’ T- and B-Lymphocyte Phenotypes and Risk for Allergic Disease

**DOI:** 10.3390/nu12103000

**Published:** 2020-09-30

**Authors:** Malin Barman, Hardis Rabe, Bill Hesselmar, Susanne Johansen, Ann-Sofie Sandberg, Agnes E. Wold

**Affiliations:** 1 Food and Nutrition Science, Department of Biology and Biological Engineering, Chalmers, University of Technology, 41296 Göteborg, Sweden; malin.barman@chalmers.se; 2 Institute of Biomedicine, Department of Infectious Diseases, University of Gothenburg, 40530 Göteborg, Sweden; hardis.rabe@gu.se (H.R.); agnes.wold@microbio.gu.se (A.E.W.); 3 Department of Pediatrics, Institute of Clinical Sciences, The Sahlgrenska Academy, University of Gothenburg, 40530 Göteborg, Sweden; bill.hesselmar@vgregion.se; 4 Pediatric Clinic, Skaraborg Hospital, 53151 Lidköping, Sweden; susanne.johansen@vgregion.se

**Keywords:** allergy, fish, fatty acids, eicosapentaenoic acid, n-3 LCPUFA, B cell, T cells, regulatory T cells, farming

## Abstract

Maternal fish intake during pregnancy has been associated with reduced allergy development in the offspring and here, we hypothesized that components of fish stimulate fetal immune maturation. The aim of this study was to investigate how maternal fish intake during pregnancy and levels of n-3 long-chain polyunsaturated fatty acids (LCPUFAs) in the infant’s cord serum correlated with different subsets of B- and T-cells in cord blood and B-cell activating factor (BAFF) in cord plasma, and with doctor-diagnosed allergy at 3 and 8 years of age in the FARMFLORA birth-cohort consisting of 65 families. Principal component analysis showed that infant allergies at 3 or 8 years of age were negatively associated with the proportions of n-3 LCPUFAs (eicosapentaenoic acid, docosapentaenoic acid, and docosahexaenoic acid) in infant cord serum, which, in turn correlated positively with maternal fish intake during pregnancy. Both maternal fish intake and cord serum n-3 LCPUFAs correlated negatively to CD5^+^ B cells and the FOXP3^+^CD25^high^ of the CD4^+^ T cell subsets in cord blood, but not to BAFF in cord plasma. Our observational study suggests that fish might contain components that promote maturation of the infant’s immune system in a manner that protects against allergy development.

## 1. Introduction

Allergy is a disease caused by an incapacity of the immune system to develop normal physiological tolerance to harmless environmental antigens, allergens. The faulty immunologic programming that leads to allergy occurs during early life, although the exact nature of this process is unknown. Several lifestyle factors are associated with a reduced risk of allergy development, including a childhood spent in poverty and under poor sanitary conditions, being part of a large family [1], and living on a farm, particularly with livestock [2,3]. Furthermore, the diet of the pregnant woman and the infant may play a role. A diet rich in fish is associated with lower risks of eczema and asthma in children, when consumed by the mother during pregnancy or during lactation [4,5,6] or by the infants themselves [7,8,9]. Certain long-chain polyunsaturated fatty acids (LCPUFAs), such as eicosapentaenoic acid (EPA, 20:5 n-3) and docosahexaenoic acid (DHA, 22:6 n-3), are only found in seafood, and the proportions of EPA and DHA in serum are markers of fish intake [10,11,12,13]. LCPUFAs are known modulators of immune functions [14,15].

We have previously shown that maternal fish intake during lactation correlates with the proportions of EPA in the breast milk of the mother and in the serum of her infant at 4 months post-partum [16]. Infant serum EPA levels, in turn, correlated negatively with the risk of being allergic at 3 years of age [16]. We have also shown in the same cohort that high proportions of circulating FOXP3^+^CD25^high^CD4^+^ T cells, suggestedly representing regulatory T cells, at birth are positively associated with being sensitized later in childhood [17] and that infants with a high proportion of CD5^+^ B cells at birth and during the first month of life are at increased risk for allergy development [18,19]. CD5^+^ B cells were mostly found to be so-called “transitional B cells”, i.e., B cells that were recently produced in the bone marrow and that had not yet undergone full maturation to become mature naïve B cells [19]. In accordance, B-cell activating factor (BAFF) was found at lower concentrations in the cord plasma of infants who subsequently develop allergy, as compared to those who did not [20].

We hypothesize that maternal fish intake during pregnancy enhances fetal immune cell maturation and thereby reduces the risk of allergy development. In the present study, we extend our previous findings by relating maternal fish intake during pregnancy to the composition of immune cells in cord blood, and with doctor-diagnosed allergy in the infants at 3 and 8 years of age. Since food intake is difficult to measure accurately, we use n-3 LCPUFAs measured in cord serum as a marker of maternal fish intake during pregnancy. The aim is to examine associations between maternal fish intake, infant immune maturation, and (protection against) allergy development.

## 2. Subjects and Methods

### 2.1. Subjects

The FARMFLORA birth-cohort study comprises 28 families who live on small, family-owned dairy farms in the Skaraborg County of southwest Sweden and 37 non-farming control families from the same rural areas [2,7,16,17,18,19,20,21,22]. Pregnant women were recruited at maternity clinics in the Skaraborg region in the period between September 2005 and May 2008. Children born at gestational weeks 36 to 42 were included. Children were followed up regularly from birth until around 8 years of age. Sixty-three children (97%) participated in the 3-year follow-up, 27 farm families and 36 non-farming control families, 48 (76%) of whom also participated in the 8-year follow-up (18 farm families and 30 non-farming control families, Table 1). The first two follow-ups were performed as close as possible to when the children turned 1.5 and 3 years, however, at the last follow-up, all children were followed-up at the same time independent of current age and therefore, the ages of the children at this follow-up varied between 6.5 and 9.4 years, with a median age of 8.3 years. The study was approved by the Regional Ethics Committee in Gothenburg (permit nos. 363-05 and 674-14) and written informed consent was obtained from both parents.

### 2.2. Clinical Examination

The children were examined clinically by one of the study pediatricians at 1.5, 3, and 8 years of age, to assess sensitization and to diagnose food allergy, eczema, asthma, and allergic rhinoconjunctivitis according to carefully standardized protocols, as described in detail below in Table 2. At 1.5 and 3 years of age, the profiles of sensitization to common foods (milk, egg, soy, fish, wheat, and peanut; six-mix food test) and inhalant allergens (birch, timothy grass, mugwort, cat, dog, horse, and house dust mite) were assessed by blood tests (Phadia, Uppsala, Sweden) [17]. At the 8-year follow-up, as previously described [18], venous blood was collected for measurements of specific immunoglobulin E (IgE) against food (milk, egg, soy, fish, wheat and peanut; six-mix food test; Phadia/Pharmacia Diagnostics, Uppsala, Sweden) and inhalant allergens (birch, timothy grass, mugwort, cat, dog, horse, and house dust mite; Phadiatop; Phadia/Pharmacia Diagnostics). Positive samples were further analyzed for specific IgE against birch, timothy, grass, mugwort, dog, cat, horse, house dust mite, cow’s milk, hen’s egg, fish, wheat, soy, and peanut (ImmunoCAP; Phadia/Pharmacia Diagnostics). An allergen-specific IgE level ≥ 0.35 kU/L was considered positive. Skin prick tests were performed against a panel of air-borne allergens (birch, grass, mugwort, cat, dog, horse, rabbit, *Dermatophagoides pteronyssinus*, *Dermatophagoides farina,* and *Cladosporium*, Soluprick SQ; ALK Abello AS, Hørsholm, Denmark) in accordance with European guidelines. Histamine (10 mg/mL) was used as the positive control and a standard allergen diluent was used as the negative control. A wheal diameter ≥ 3 mm was considered positive. Sensitization was defined as a positive Phadiatop test or a positive 6-food mix test (ImmunoCAP ≥ 0.35 kU/L).

Allergy in the children was defined as having one or more of: allergic rhinoconjunctivitis (ARC), asthma, eczema, and food allergy. Diagnoses of ARC, asthma, eczema, and food allergy at 1.5, 3, and 8 years of age were made according to strict criteria (Table 2).

### 2.3. Dietary Assessment

Food intake during pregnancy was reported shortly after delivery using a semi-quantitative food-frequency questionnaire (FFQ) adapted from the Northern Sweden 84-item questionnaire [26], as extended with food items reflecting fat-containing foods [27]. The FFQ was returned by 21 Farm mothers and 36 non-Farm mothers (Table 1). Frequencies were reported on a nine-level scale: never/seldom, once a month, twice a month, once a week, 2–3 times a week, 4–6 times a week, once a day, 2–3 times a day, and ≥4 times a day. The frequencies were converted to intake in grams per day, based on reported portion sizes, or when not reported, standard portion sizes according to the Swedish National Food Agency (2001).

### 2.4. Analysis of Fatty Acids in Cord Sera

Cord blood serum was collected from 54 of the infants at the time of delivery (Table 1). The samples were centrifuged, and the sera were immediately frozen in aliquots and stored at −80 °C until analyses. The total fatty acid composition was determined for cord serum samples using gas chromatography-mass spectrometry (GC-MS) after the extraction and conversion of the fatty acids to methyl esters according to the method of Lepage and Roy [28]. Briefly, serum (100 µL aliquot) was mixed with an internal standard C17:0 (5 µg), followed by the addition of 2 mL toluene and 2 mL acetyl chloride dissolved in methanol (10% v/v). The fatty acids were converted to methyl esters in a 70 °C water-bath for 2 h and thereafter extracted with petroleum ether, evaporated under a stream of N_2_ gas, dissolved in 200 µL isooctane, and transferred to vials. Each serum sample was prepared and analyzed in duplicate. The fatty acids were separated using gas chromatography (Agilent 7890A; Agilent Technologies, Santa Clara, CA, USA) on a VF-WAXms (30 m × 0.25 mm × 0.25 µm) column (Agilent Technologies), and quantified by mass spectrometry (5975C inert XL EI/CI MSD with Triple-Axis Detector; Agilent Technologies) after electron ionization. The fatty acids were separated at an initial temperature of 100 °C, with an increase in temperate to 205 °C at the rate of 4 °C/min, and thereafter with an increase of 1 °C/min up to 230 °C, which was held for 5 min. The total run time was 56 min per sample. Helium was used as the carrier gas. Data were acquired using the MSD Chemstation (Agilent Technologies, Santa Clara, CA, USA). The methylated fatty acid mixture GLC 364 (Nu-Chek-Prep Inc., Elysian, MN, USA) was used as standard for identification of the different peaks. Eighteen fatty acids were quantified in each sample: 14:0, 16:0, 16:1 n-7, 18:0, 18:1 n-7, 18:1 n-9, 18:2 n-6, 18:3 n-3, 20:0, 20:3 n-6, 20:4 n-6, 20:5 n-3, 22:0, 22:4 n-6, 22:5 n-3, 22:5 n-6, 22:6 n-3, and 24:0. The proportions of specific fatty acids are expressed as the area of the peak of the particular fatty acid relative to the total peak area of all 18 fatty acids.

### 2.5. Flow Cytometry Analysis of Immature, Naive, and Memory Lymphocyte Markers in Cord Blood

The proportions of different subsets of lymphocytes were analyzed using flow cytometry within 72 h of the cord blood samples being obtained (*n* = 48), as previously described [29,30]. In brief, whole blood was incubated for 20 min at 4 °C with the anti-human monoclonal antibodies described in Supplementary Materials Table S1. Red blood cells were then lysed (FACS lysing solution; BD Bioscience, Erembodegem, Belgium) and the remaining cells were washed twice with FACS-buffer. After completion of cell surface staining, the cells were intracellularly stained for FOXP3 using the PE anti-human FOXP3 Staining Set (eBioscience, San Diego, CA, USA). All isotype controls were purchased from BD Bioscience, except for the isotype control for FOXP3 that was purchased from eBioscience. As described in detail previously, the total cell counts of lymphocytes, B cells, and CD4^+^ T cells were determined with the Trucount assay (BD Biosciences) [20]. Stained samples were analyzed in a FACS Calibur (BD Bioscience) equipped with the CellQuestPro software. Data were examined using the FlowJo software (TreeStar, Ashland, OR, USA). The gating strategy applied has been described previously in detail [29,31].

### 2.6. Analysis of B Cell Activating Factor in Cord Plasma

The analysis of B cell activating factor (BAFF) levels has been previously described [20]. Plasma was isolated from cord blood samples at birth (*n* = 58) and stored at −80 °C until analysis. The concentrations of BAFF were determined using BAFF/BLyS/TNFSF13B Quantikine ELISA Kits (R&D Systems, Minneapolis, MN, USA) according to the manufacturer’s protocol. In short, plasma samples were diluted 1:2 with Calibrator Diluent RDQ and added to ELISA plates pre-coated with monoclonal antibodies towards BAFF. The ELISA plates were incubated for 3 h with diluted plasma samples, 1 h with Human BAFF/BLys conjugate, and 30 min dark with substrate. All incubations were performed at room temperature and the plates were washed 3 times with wash buffer in between each incubation. The substrate reaction was stopped with stop solution and the optical density was determined at a wavelength of 450 nm.

### 2.7. Statistical Methods

Participant characteristics were analyzed using the χ^2^ test or Fisher’s exact test for categorical variables and the Mann–Whitney *U*-test for continuous variables. Differences in the levels of serum fatty acids were analyzed using the Mann-Whitney *U*-test. Correlations were calculated by Spearman’s rho. Two-tailed *p*-values ≤ 0.05 were considered statistically significant. Logistic regression was used to analyze effect of cord serum EPA proportions on allergy. Odds ratios (OR) were presented per standard deviation (SD) of EPA proportions, i.e., 0.18%. Principal Component Analysis (PCA) was used to display the distribution of the subjects in the multivariate space based on the distribution of immune cells in umbilical cord blood and BAFF in the umbilical cord plasma at birth, and proportions of fatty acids in the umbilical cord serum from the children, maternal intake of fish during pregnancy, and the presence of allergy in the children at 3 and 8 years of age. Orthogonal projection to latent structures (OPLS) was used to investigate which of the variables were most strongly related (positively or negatively) to an allergy diagnosis at 3 or 8 years of age. To visualize further the associations between immune cells in umbilical cord blood, and BAFF in the cord plasma, fatty acids in cord serum, and fish intake by the mother, unsupervised hierarchical cluster analyses were conducted using Spearman’s correlations and presented in heatmaps, together with partial Spearman’s correlations. The cluster analysis automatically structures the variables and places correlated variables next to each other. The analyses were performed with the following software packages: IBM SPSS ver. 26 (IBM, New York, NY, USA), R ver. 3.6.2 (Vienna, Austria), and Umetrics SIMCA ver. 16.0.1 (Sartorius Stedim Data Analytics AB, Umeå, Sweden).

## 3. Results

### 3.1. Characteristics of the Study Population

The infants in the FARMFLORA cohort were followed up regarding allergy at 1.5, 3, and 8 years of age. The allergy distribution has previously been reported in detail [2,17,18,20]. In brief, 64, 63, and 48 infants participated in the follow-ups at 1.5, 3, and 8 years respectively, and 15, 11, and 10 infants were diagnosed as allergic (i.e., having one or more of allergic rhinoconjunctivitis (ARC), asthma, eczema, and food allergy) (Table 3).

At 1.5 years of age, eczema was found in 12 (19%) of the 64 infants, asthma was found in 5 (8%), ARC in 2 (3%), and food allergy in 2 (3%) of the infants. At 3 years of age, 7 (11%) children had eczema, 4 (6%) had asthma, 1 (2%) had ARC, and 2 (3%) had food allergy. At 8 years of age, 5 (19%) had eczema, 4 (8%) had asthma, 3 (6%) had ARC, and none had food allergy [18]. Infants who were allergic at 1.5 years but were not allergic at 3 years of age (“transient allergy”) were excluded from the non-allergic group at 3 years (*n* = 7). Likewise, the infants who were allergic at 1.5 or 3 years but not allergic at 8 years of age were not included as non-allergic controls at 8 years of age (*n* = 4).

At 3 years of age, allergy was significantly more prevalent in the non-farming control group than among farmers’ children, while this difference had disappeared at 8 years of age (Table 4). Allergic and non-allergic infants at 3 and 8 years of age were similar regarding parental heredity, maternal education, maternal and paternal smoking, cat- and dog-keeping, having elder siblings, gestational length, delivery by Cesarean section, birthweight, and male gender (Table 4 and partly reported previously [7,18]).

### 3.2. Cord Serum LCPUFA Pattern Reflects Maternal Intake of Seafood during Pregnancy

**Figure 1:**
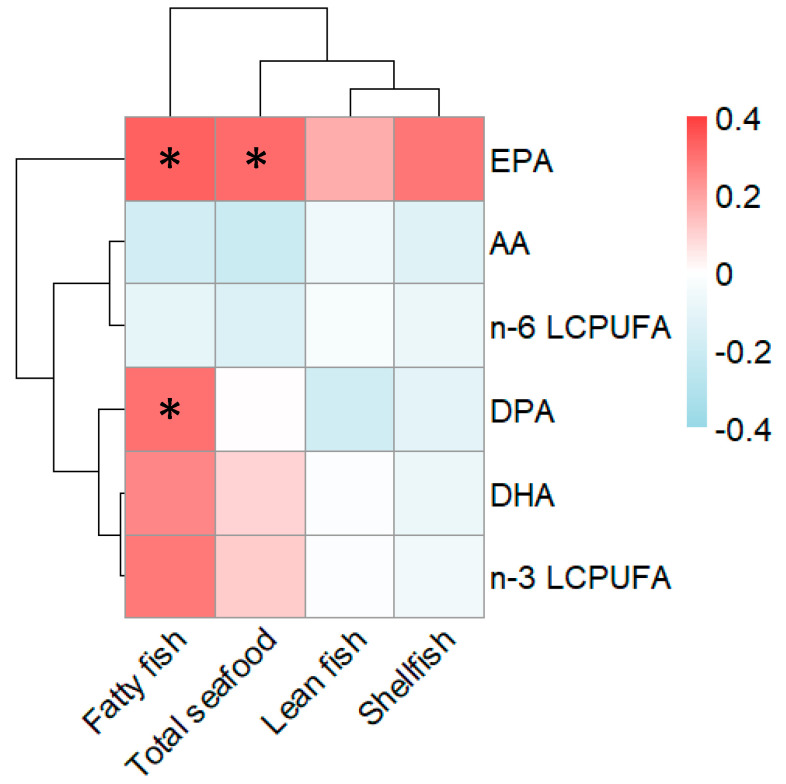
Heatmap of the correlations between reported maternal seafood intake during pregnancy and proportions of fatty acids in cord serum. The magnitude of each correlation is denoted with a color, whereby the red color indicates a positive correlation and the blue color represents a negative correlation. A darker shade of each color indicates a stronger correlation. Statistical significance using Spearman correlations is denoted by an asterisk (*p* < 0.05). AA, Arachidonic acid; EPA, eicosapentaenoic acid; DPA, docosapentaenoic acid; DHA, docosahexaenoic acid; LCPUFA, long-chain polyunsaturated fatty acid.

First, we investigated whether a maternal diet rich in fish would influence the fetal milieu in which the immune system develops. The focus here was on LCPUFAs, which are known modulators of immune function and exclusively found in seafood, i.e., lean and fatty fish and shellfish. Correlations between the proportions of relevant LCPUFAs in the infant cord serum and the mother’s reported intake of different types of seafood during pregnancy are shown in a heatmap in Figure 1.

As shown in Figure 1, EPA as a proportion of the total amount of fatty acids in the infants’ cord serum correlated positively with the mothers’ reported intake of fatty fish during pregnancy (Rho = 0.33, *p* = 0.02), as well as with the total intake of seafood (Rho = 0.31, *p* = 0.03). Also, the proportion of docosapentaenoic acid (DPA, 22:5 n-3) in cord serum was significantly (Rho = 0.30, *p* = 0.04) related to maternal intake of fatty fish, though not to maternal intake of total seafood. Thus, EPA and DPA measured in cord serum could serve as biomarkers, validating the pregnant woman’s intake of seafood, with the latter reflecting mainly the intake of fatty fish. It is noteworthy that the proportion of DPA, DHA, and total n-3 LCPUFAs clustered together in the heat map, suggesting that they are related to each other, while EPA did not cluster together with the other n-3 LCPUFAs. As expected, the proportion of the n-6 LCPUFA arachidonic acid (AA) and the total n-6 LCPUFAs in cord serum were unrelated to maternal fish intake (Figure 1).

### 3.3. Relationships between Maternal Intake of Fish, Cord Blood Immune Profile, and Allergy at 3 and 8 Years of Age

**Figure 2:**
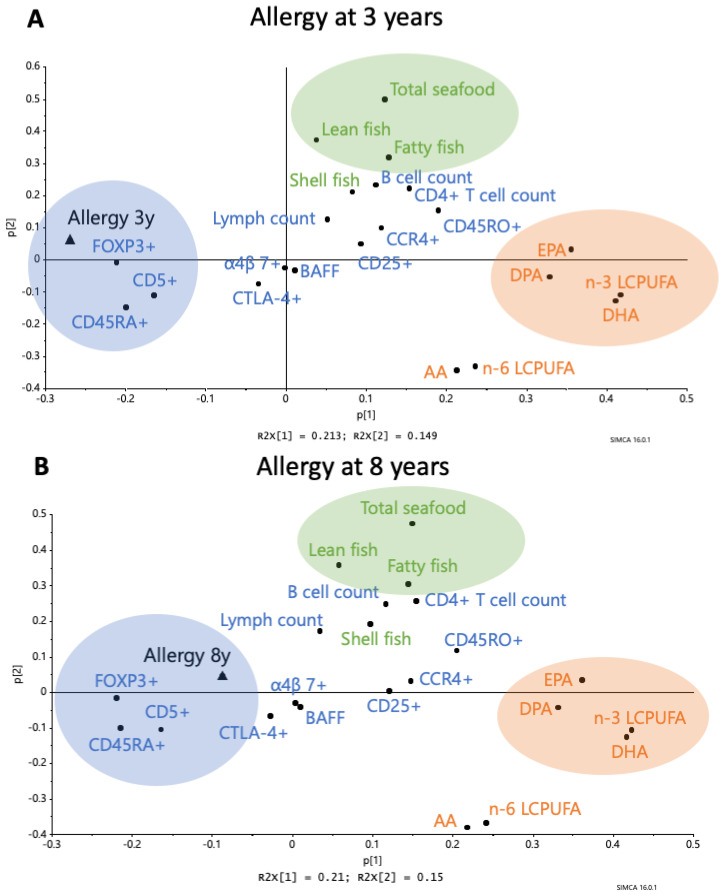
Principal Component Analysis (PCA) loading plot showing the associations between maternal fish intake during pregnancy, LCPUFAs (EPA, DHA, total n-3 LCPUFAs, AA, and total n-6 LCPUFAs) in the cord serum, circulating B cells (B cell count, CD5^+^) and CD4^+^ T cells (CD4^+^ T cell count, activated CCR4^+^, activated CD25^+^, memory CD45RO^+^, naïve CD45RA^+^, naïve α4β7^+^, CTLA4^+^CD25^+^, FOXP3^+^CD25^high^) in umbilical cord blood, and B cell activating factor (BAFF) in the cord plasma and allergy at 3 years of age (**A**) and 8 years of age (**B**). Abbreviations: 3y, 3 years; 8y, 8 years, AA, arachidonic acid, EPA, eicosapentaenoic acid; DPA, docosapentaenoic acid; DHA, docosahexaenoic acid; LCPUFA, long-chain polyunsaturated fatty acids. All 65 infants in the FARMFLORA cohort were included in the models.

The relationships between maternal fish intake during pregnancy, LCPUFAs in the cord serum, and immune parameters in cord blood at birth were explored using PCA. In the first loading plot, these variables were related to allergy at 3 years and in the second plot with allergy diagnosed at 8 years of age (Figure 2A,B). In the PCA loading plots, along the first component, the proportions of different n-3 LCPUFAs in cord serum appear at the opposite end of the diagram to being allergic at 3 years of age (Figure 2A) or 8 years of age (Figure 2B). From the heatmap in Figure 1, it is clear that the n-3 LCPUFAs EPA and DPA in cord serum samples are the fatty acids that appear closest to the maternal fish intake. DHA and n-3 total LCPUFAs (reflecting mainly DHA, since DHA constitutes the major proportion of the total LCPUFAs) appear slightly further from the maternal dietary fish consumption (Figure 2A,B).

The immune parameters that showed up close to the “allergy” variable were: (1) a high proportion of cord blood B cells expressing the marker CD5, and (2) a high proportion of the CD4^+^ T cells expressing the naivety marker CD45RA^+^ or being FOXP3^+^CD25^high^ (Figure 2A,B). This was true whether allergy was diagnosed at 3 or 8 years of age (Figure 2A,B). In contrast, markers of immune maturation, such as the number of CD4^+^ T cells/mL blood and the proportion of T cells expressing the memory marker CD45RO or the homing receptor CCR4, appeared furthest away from the allergy diagnosis at either 3 or 8 years of age (Figure 2A and B, respectively).

### 3.4. Factors Relating to Allergy Diagnosis at 3 or 8 Years of Age

**Figure 3:**
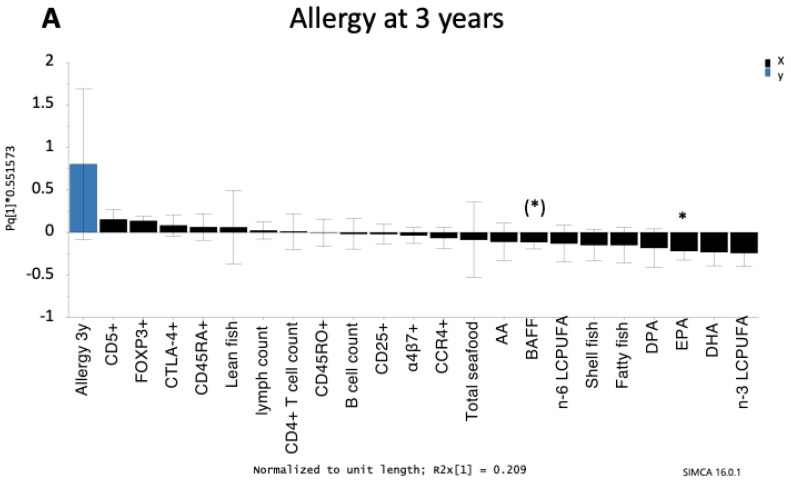

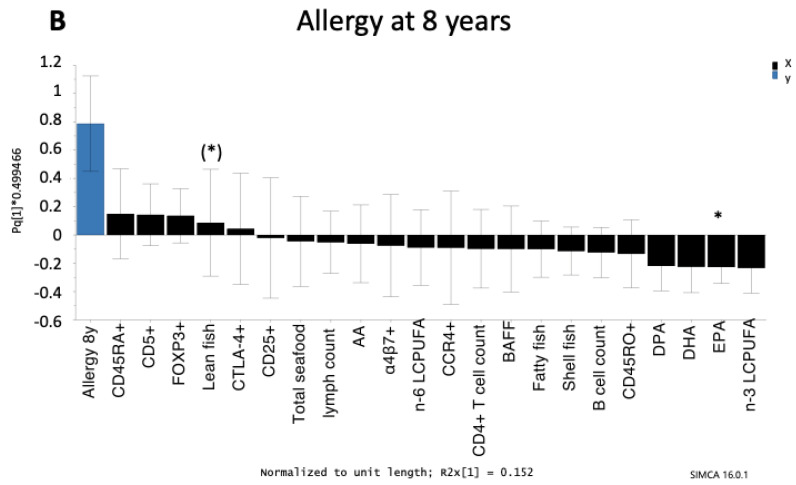
Orthogonal projection to latent structures (OPLS) loading column plot showing the associations between allergy at 3 years of age (**A**) and 8 years of age (**B**) and maternal fish intake during pregnancy, LCPUFAs (% of total fatty acids), levels of B cell activating factor (BAFF) in cord serum, and circulating B cells (B cell count/mL and immature CD5^+^) and CD4^+^ T cells (CD4^+^ T cell count/mL, CCR4^+^, CD25^+^, memory CD45RO^+^, naïve CD45RA^+^, naïve α4β7^+^, CTLA4^+^CD25^+^, FOXP3^+^CD25^high^) in cord blood. Only those infants with an allergy diagnosis at 3 or 8 years of age were included in the models. Children who were diagnosed with an allergic disease at an earlier age but were healthy at 3 or 8 years of age (transient allergy) were excluded. Thus, 55 infants were included in the OPLS model for allergy at 3 years of age and 44 infants were included in the OPLS model for allergy at 8 years of age. Associations that were significant in univariate analyses (Mann–Whitney *U*-test) are indicated with an asterisk: ^(^*^)^ *p* < 0.1, * *p* < 0.05. Abbreviations: 3y, 3 years; 8y, 8 years; AA, arachidonic acid; BAFF, B cell activating factor; EPA, eicosapentaenoic acid; DPA, docosapentaenoic acid; DHA, docosahexaenoic acid; LCPUFA, long-chain polyunsaturated fatty acids.

Next, we used OPLS models to uncover which of the variables were most strongly related (positively or negatively) to an allergy diagnosis at 3 or 8 years of age. Allergy was set as the Y-variable, and maternal fish intake, cord serum fatty acids, and immune parameters in cord blood were set as explanatory variables (Figure 3A,B). Univariate statistics were performed to confirm that the associations were statistically significant and are designated by asterisks in the OPLS plots. We found that allergy at both 3 and 8 years of age was negatively associated with the levels of EPA in cord serum (*p* = 0.018 and *p* = 0.029, respectively) (Figure 3A,B). Although allergy at 3 years of age was inversely associated with BAFF levels in the OPLS analysis (Figure 3A), this association was not significant in the univariate analysis (*p* = 0.06). Similarly, the observed association between allergy at 8 years of age and higher intake of lean fish was not significant in the univariate analysis (*p* = 0.09) (Figure 3B).

It should be pointed out that although allergy was diagnosed at both 3 and 8 years of age in the same birth-cohort, those who were allergic at both time-points were not necessarily the same children. Of the eleven children who were allergic at 3 years of age, five were still allergic at 8 years of age, four had outgrown their allergy, and two were not followed-up at 8 years. Of the ten children who were allergic at 8 years of age, four were allergic at both 1.5 and 3 years of age, two were allergic at 1.5 years of age but non-allergic at 3 years of age, one was allergic at 3 years of age but not at 1.5 years of age, and three were non-allergic at both 1.5 and 3 years of age. Despite this, the immune and dietary factors that were associated (positively or negatively) with allergy were largely the same.

### 3.5. Magnitude of Putative Allergy-Preventive Effect of Maternal Fish Intake

Table 5 shows the maternal fish intake during pregnancy in relation to child allergy status at 8 years of age. Maternal fish intake in relation to child allergy at 3 years of age have previously been published [2]. Table 6 shows the median proportion of LCPUFAs in cord serum in relation to allergy at 3 and 8 years of age. For proportions of all fatty acids in cord serum, see Supplementary Materials Table S2.

The fish biomarker EPA in cord serum was negatively related to allergy at both 3 and 8 years of age (Table 6). We used logistic regression to calculate how much an increment in one standard deviation of cord serum EPA (0.18%) would reduce the risk of an infant becoming allergic. For allergy at 3 years of age, the Odds Ratio (OR) was 0.20 (95% confidence interval (CI) 0.04–0.97; *p* = 0.045) per standard deviation (SD) of EPA. For allergy at 8 years of age, the corresponding OR was 0.19 (95% CI 0.03–1.3; *p* = 0.088).

### 3.6. Maternal Seafood Diet, Cord n-3 LCPUFA, and Immune Parameters in the Newborn Infant

**Figure 4:**
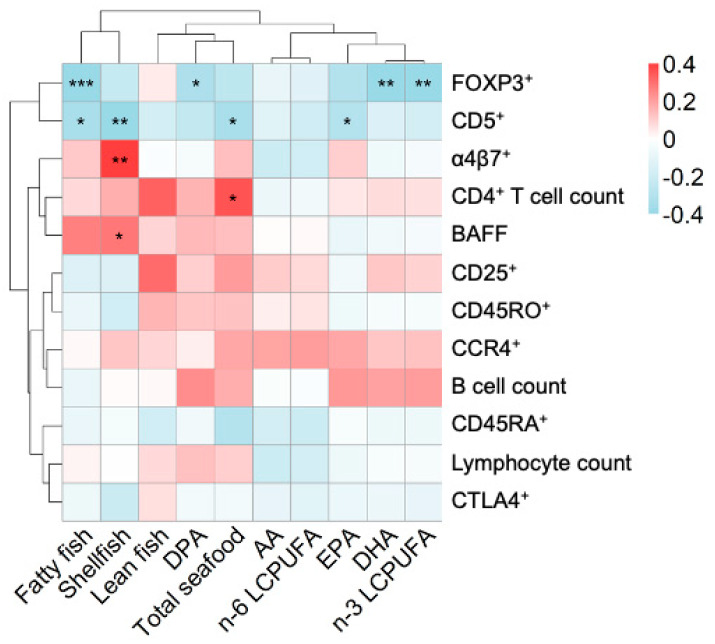
Heatmap showing the correlations between reported maternal seafood intake during pregnancy, fatty acids in cord serum, and immune cells in the cord blood. The magnitude of each correlation is indicated with a color, whereby the red color indicates a positive correlation and the blue color represents a negative correlation. A darker shade of each color indicates a stronger correlation. Levels of statistical significance using Spearman correlations are denoted as: * *p* < 0.05, ** *p* < 0.01, *** *p* < 0.001. Abbreviations: AA, arachidonic acid; EPA, eicosapentaenoic acid; DPA, docosapentaenoic acid; DHA, docosahexaenoic acid; LCPUFA, long-chain polyunsaturated fatty acid; CD25, percentage of CD25^+^ cells in CD4^+^ T-cell population; CCR4, percentage of CCR4 cells in CD4^+^ T-cell population; a4b7, percentage of α4β7^+^ cell in the of CD4^+^ T-cell population; CD45RO, percentage of memory CD45RO^+^ cells in the CD4^+^ T-cell population; FOXP3, percentage of FOXP3^+^CD25^high^ cells in the CD4^+^ T-cell population; CD45RA, percentage of naive CD45RA^+^ cells in the CD4^+^ T-cell population; CTLA4, percentage of CTLA-4^+^CD25^+^ cells in the CD4^+^ T-cell population; BAFF, levels of B cell activating factor.

We hypothesized that components of fish in the maternal diet affect fetal immune maturation. To investigate this, we generated a heatmap of the correlations between maternal seafood intake and levels of cord serum LCPUFAs on the one hand, and a range of cellular and plasma immune parameters in infant cord blood on the other hand. In the upper part of Figure 4, the proportion of B cells expressing CD5 (“transitional” B cells) is grouped together with the proportion of CD4^+^ T cells that is FOXP3^+^CD25^high^. A high proportion of CD5^+^ B cells has previously been shown to be associated with subsequent allergy development [18,19], while a high proportion of CD4^+^ T cells having the FOXP3^+^CD25^high^ phenotype is associated with a high risk of subsequent sensitization to common environmental allergens [17]. Interestingly, both these variables correlated negatively with maternal seafood intake and infant cord serum n-3 PUFAs of the types found in seafood. The proportion of FOXP3^+^CD25^high^CD4^+^ T cells in cord blood correlated negatively with the proportion of cord serum n-3 LCPUFAs, including DHA and DPA, although not with the proportion of EPA, and also correlated very strongly and negatively with the maternal intake of fatty fish (Rho, −0.57; *p* < 0.001) (Figure 2). Maternal intake of fatty fish, shellfish, and total seafood also correlated negatively with the population of immature CD5^+^ B cells, which is a cell population that we have previously found to be positively related to allergy development (Figure 2).

Other markers that grouped together and were instead positively related to maternal seafood intake were: BAFF, the numbers of CD4^+^ T cells in cord blood, and the proportion of CD4^+^ T cells expressing the gut homing marker α4β7^+^. These variables were all related to maternal intake of seafood, albeit not to the intake of particularly fatty fish, but rather a diet rich in lean fish and shellfish (Figure 4). Notably, a majority of the naive T lymphocytes in the cord blood express the gut homing marker α4β7^+^ [30,31], so maternal fish intake may correlate with a high proportion of naïve T cells in cord blood, produced from the thymus during fetal life.

Lastly, variables relating to T-cell activation and maturation grouped together in the middle of the heatmap in Figure 4 (expression of the activation marker CD25, the memory marker CD45RO, and the homing marker CCR4 of CD4^+^ T cells), together with the B cell counts. All of these variables were weakly related to maternal fish intake or infant cord serum LCPUFA pattern.

## 4. Discussion

The FARMFLORA birth-cohort was designed to study aspects of farm living, including dietary intakes typical of farming families, as protective factors against allergy in childhood. Farming families consume more saturated fats, while non-farming families consume more unsaturated fats. We have shown that during pregnancy and lactation, a maternal diet that is rich in margarine and oils is linked to a higher risk of allergy in the offspring [2]. However, unrelated to the farming environment, we have previously found a negative association between EPA in infant serum at 4 months and allergy in the child at 3 years of age in this cohort [16]. The proportions of EPA in the sera of infants at 4 months of age were linked to maternal breast milk proportions of EPA which, in turn, were related to maternal intake of fatty fish at 4 months postpartum [16]. In the present study, we extend these previous findings by including allergy diagnosed at 8 years of age in the same cohort, and by relating maternal fish intake during pregnancy and the LCPUFA levels in infant cord serum to several cellular and soluble immune parameters in the same cord blood samples. The aim is to disentangle the complex web of dietary exposures, immune maturation, and (protection against) allergy development.

We showed that allergy diagnosed at either 3 or 8 years of age is clearly inversely related to the proportion of EPA in the cord serum of the infant, with ORs of 0.20 and 0.19 respectively, for each increase in one standard deviation of EPA (proportions of total fatty acids) in the cord serum. EPA is an n-3 LCPUFA that is exclusively present in seafood and is a recognized marker of fish intake [10,11,12,13]. Here, we showed that the proportions of EPA in the cord serum of the newborn infant correlate with the mother’s reported intake of both fatty fish and total seafood during pregnancy.

We attribute the negative association between cord serum EPA and subsequent allergy in the child to a protective effect of maternal seafood intake, rather than to an effect of EPA *per se*. Several studies have pointed to a protective effect of maternal fish intake on childhood allergy [4,5,6]. That we could not demonstrate a significant protective effect of maternal fish intake at either 3 or 8 years of age (univariate analysis) may reflect the small size of the cohort and the difficulties associated with accurately measuring food intake using food frequency questionnaires [32,33,34]. EPA is a recognized biomarker of fish intake [10,11,12,13] and it may be well suited to serving as a proxy for maternal fish intake.

DHA is the main long-chain fatty acid in fish and shellfish. Interestingly, the proportions of DHA in infant cord serum correlated only weakly to the mother’s intake of fatty fish, and not to the intake of other types of seafood, while the cord serum EPA levels were significantly related to fatty fish and total seafood consumption by the mother. This discrepancy in correlation patterns between EPA and other n-3 LCPUFAs may be explained by the differences in efficiencies with which these fatty acids are transported between the maternal and the fetal circulation during pregnancy. There is selective and active transport of LCPUFAs across the placenta, mediated by specific transport proteins, with prioritization of those fatty acids that are specifically needed by the fetus [35]. As DHA is needed for brain growth, it is transported against its concentration gradient to ensure that the fetus receives sufficient DHA for growth [35]. EPA is prioritized to a lesser extent in the placental transport from the mother to the fetus and may, therefore, be a better marker of available maternal fatty acid proportions and consequently, maternal fish intake. We have previously compared the fatty acid proportions in the cord sera of children born to farming and non-farming mothers and have shown that the children born to farming mothers have higher proportions of arachidonic acid and another n-6 LCPUFA, adrenic acid, in their cord serum than children born to non-farming mothers [22]. However, none of these n-6 LCPUFAs correlated with immune biomarkers or allergy in the offspring [22].

The detailed analysis of soluble and cellular immune parameters in blood samples obtained at delivery provides some clues as to the possible influencing factors through which intake of fish may affect beneficially healthy immune maturation and prevent or counteract allergy development. We showed that maternal intake of fatty fish, shellfish, and total seafood during pregnancy, as well as the cord serum levels of the fish biomarker EPA, correlate negatively with the proportions of cord blood B cells that express the marker CD5 (mainly representing very immature B cells), termed “transitional B cells”, and previously shown to be a predictor of allergy development in this cohort [19]. The levels of BAFF, which drives B-cell maturation and activation, related positively to maternal intake of shellfish during pregnancy. BAFF was previously shown to be protective against allergy development in this cohort, and the BAFF levels in cord plasma were also higher in newborn infants from farming versus non-farming families [20]. The cord plasma levels of BAFF tended to be negatively associated with allergy also in the analysis made here, although the differences did not reach statistical significance. This discrepancy may be due to the fact that only part of the cohort was included in the published paper [20], whereas more infants are included in the current study. Taken together, our results suggest that factors that increase B-cell maturation during fetal life are protective against allergy development. The effects on infant B-cell parameters correlate with the levels of maternal fish intake and cord serum EPA, but not with the levels of other n-3 LCPUFAs found in fish, such as DHA or DPA, or with total n-3 LCPUFAs. Similarly, maternal consumption of fatty fish is not related to infant B-cell maturation parameters. One can speculate that components of fish other than n-3 LCPUFAs provide B-cell-stimulating signals. Fish are rich in proteins, vitamins B12, A and D, iodine, zinc, iron, and selenium, any of which could affect fetal B-cell maturation.

With respect to T cells, we observed that the proportion of infant cord blood CD4^+^ T cells that expressed the marker of naivety CD45RA or that had the FOXP3^+^CD25^high^ phenotype, regarded as a marker of regulatory T cells, were positively associated with allergy, in the PCA as well as in the OPLS, although the differences between these variables did not reach statistical significance in the univariate analysis. We have previously found, in the same cohort, that higher proportions of CD4^+^ T cells of the FOXP3^+^CD25^high^ phenotype at birth and at 3 days of age are positively associated with sensitization, i.e., the production of IgE antibodies against environmental allergens, at 3 years of age [17]. High proportions of regulatory T cells early in life are associated with delayed activation of the adaptive immune response and can result in prolongation of the immune immaturity that in theory can pave the way for allergy development [32]. Accordingly, preterm children have higher proportions of FOXP3^+^CD25^high^ CD4^+^ T cells than children who are born at term [36,37]. In the present study, this T-cell population is negatively associated with n-3 LCPUFA levels in the cord serum, including the levels of DHA and DPA, and is also negatively associated with the reported consumption of fatty fish by the pregnant woman. Thus, our results suggest that consumption of fatty fish during pregnancy stimulates fetal T-cell maturation, perhaps mediated by the immunomodulating n-3 PUFAs. It is noteworthy that for this marker of T-cell immaturity, there is no correlation with either lean fish consumption or total seafood consumption.

We also found that maternal total seafood intake is positively related to the total count of CD4^+^ T cells in cord blood, and shellfish consumption is related to the proportion of CD4^+^ T cells expressing the gut homing marker α4β7. The integrin α4β7 is expressed by 90% of CD4^+^ T cells and binds to its ligand MAdCam-1 (mucosal vascular addressin cell adhesion molecule-1), which is normally expressed in the gut. In fetuses and newborn children, however, MAdCam-1 can be detected in the peripheral lymph nodes [38], which increases the possibility for naïve T cells to encounter the target antigen and be activated.

A major limitation of the present study is the small sample size. Nevertheless, the size of the study cohort permitted thorough clinical examinations of the children by a pediatrician at 1.5, 3, and 8 years of age, as well as between follow-ups when symptoms suggesting the commencement of allergic disease were noted. Furthermore, all the diagnoses were based on strict criteria. When defining the allergic and the non-allergic groups at 3 and 8 years of age, infants who were allergic at an early age but not allergic at the subsequent follow-up (“transient allergy”) were not included in any of the groups. Another strength of this study is the application of biomarkers of fish intake.

## 5. Conclusions

Our results suggest that fish contain components that promote the maturation of the infant immune system in a manner that protects against allergy development, thereby confirming the previous finding of a negative association between maternal fish diet during pregnancy and allergy development in the child [4,5,6]. Our results also suggest that certain immune parameters are primarily affected by the LCPUFA content of the fish, while others may be more related to fish consumption per se and may be affected by any type of bioactive substance in the fish.

## Figures and Tables

**Table 1:** Number of subjects involved in different parts of the study.

	Total	Farm Families	Non-Farm Families
Number of enrolled families	65	28	37
Participated in the 1.5 years follow-up	65	28	37
Participated in the 3 years follow-up	63	27	36
Participated in the 8 years follow-up	48	18	30
Fatty acid analyses in cord serum	54	25	29
Flow cytometry analyses on cord blood	47	21	26
BAFF analyses on cord plasma	58	25	33
Maternal dietary data collected	57	21	36

Abbreviations: BAFF, B cell-activating factor.

**Table 2:** Diagnostic criteria for airway rhinoconjunctivitis (ARC), asthma, eczema, and food allergy at 1.5, 3, and 8 years of age in the FARMFLORA study.

Age of Child	ARC	Asthma	Eczema	Food Allergy
1.5 years	Symptoms from eyes and/or nose on exposure to pollen or animals with positive IgE/SPT to respective allergen	≥3 episodes of wheezing in the previous year plus any of: - symptoms outside infections - other clinical manifestations of allergy or persistent wheeze with duration ≥ 4 weeks	Itching spots at typical locations that have come and gone for at least 6 months or based on William’s criteria [23,24,25]	An immediate- or late-onset reaction after ingestion of the specific food, followed by a distinct and prompt clinical improvement when eliminating the suspected food allergen, and any of: - other signs of allergic disease - more than one organ system involved - supported by positive allergy tests, biopsies, or challenge tests
3 years	Same as 1.5 years, with symptoms during last 12 months	≥3 episodes of wheezing, with the last episode after 2 years of age, plus any of: - symptoms outside infections - other allergic manifestations or wheezing with onset after 2 years of age, and any instances of wheezing: - triggered by colds in children with other manifestations of allergy - triggered by exercise - response to anti-inflammatory maintenance therapy or persistent wheeze with duration ≥ 4 weeks in the previous year	Same as 1.5 years, with symptoms during last 12 months	Same as 1.5 years, with symptoms during last 12 months
8 years	Same as 1.5 years, with symptoms during last 12 months	Wheeze/heavy breathing and any of: - response to anti-inflammatory maintenance therapy - Bronchial hyperreactivity with PD20 < 0.6 mg methacholine - FEV_1_reversibility > 12%	Same as 1.5 years, with symptoms during last 12 months	Symptoms of food allergy, supported by open food challenge, either planned or accidental

ARC, Allergic rhinoconjunctivitis; IgE, immunoglobulin E; SPT, skin prick test; FEV_1_, forced expiratory volume in 1 s; PD20, provocative dose causing a 20% fall in FEV_1_.

**Table 3:** Allergy distribution in the FARMFLORA study.

	Total	Farm Group	Non-Farm Group
Number of enrolled families	65	28	37
Any allergy at 1.5 years of age	15	2	13
Any allergy at 3 years of age	11	1	10
Any allergy at 8 years of age	10	3	7

Any allergy: having one or more of allergic rhinoconjunctivitis (ARC), asthma, eczema, and food allergy. The information in the table have previously been reported [2,17,18,20].

**Table 4:** Differences in background characteristics between allergic and non-allergic infants in the FARMFLORA cohort.

Variable	Non-Allergic at 3 Years of Age (*n* = 44)	Allergic at 3 Years of Age (*n* = 11)	*p*	Non-Allergic at 8 Years of Age (*n* = 34)	Allergic at 8 Years of Age (*n* = 10)	*p*
	*n* (%) or median (25–75 percentile)			*n* (%) or median (25–75 percentile)		
Farm family	23 (52%)	1 (9%)	**0.02**	15 (40%)	3 (30%)	0.72
Maternal heredity ^a^	9 (21%)	5 (46%)	0.12	7 (21%)	4 (40%)	0.21
Paternal heredity ^a^	7 (16%)	3 (27%)	0.40	7 (21%)	3 (30%)	0.53
Maternal education, level ^b^	4 (1–5)	3 (1–5)	0.78	4 (2–5)	2 (2–5)	0.21
Maternal smoking ^c^	1 (2%)	0 (0%)	1.00	1 (3%)	0 (0%)	0.58
Paternal smoking ^c^	2 (5%)	2 (18%)	0.18	2 (6%)	0 (0%)	0.42
Dogs in house at recruitment	17 (39%)	1 (9%)	0.08	12 (35%)	1 (10%)	0.24
Cats in house at recruitment	21 (48%)	4 (36%)	0.50	14 (41%)	4 (49%)	1.00
Siblings	23 (52%)	6 (55%)	0.89	18 (53%)	3 (30%)	0.20
Gestational week	39 (36–42)	39 (36–41)	0.14	39 (38–40)	40 (37–40)	0.97
Cesarean section	5 (11%)	4 (36%)	0.07	4 (12%)	4 (40%)	0.06
Birth weight, grams	3560 (2440–4740)	3235 (2730–4830)	0.14	3598 (3245–3928)	3288 (3071–3821)	0.29
Male gender	22 (50%)	9 (82%)	0.09	14 (41%)	6 (60%)	0.23

Differences between allergic and non-allergic individuals were analyzed with Chi-square test or Fishers exact test for categorical data and Mann–Whitney *U*-test for continuous data. Significant differences are marked in bold. ^a^ Doctor-diagnosed asthma, rhinitis, or atopic eczema. ^b^ 1 = lowest, 5 = highest, 1 = elementary school, 2 = upper secondary school for two to three years or equivalent, 3 = qualified graduate from upper secondary engineering course, 4 = university ≤ 1 year, 5 = university > 1 year. ^c^ Maternal or paternal smoking during last month of pregnancy. Data presented in this table has partly been published before [7,18].

**Table 5:** Maternal intake of fish during pregnancy in relation to allergy diagnosis at 8 years of age.

Food Intake, Grams per Day	Non-Allergic at 8 Years of Age (*n* = 25)	Allergic at 8 Years of Age (*n* = 8)	*p* ^a^
Fatty fish	8 (0.0–10)	3.1 (0.0–12)	0.492
Lean fish	11 (5.4–16)	21 (11–21)	0.084
Shellfish	3.3 (0.0–6.4)	0.0 (0.0–4.8)	0.092
Total seafood intake	25 (11–30)	25 (13–37)	0.737

Data are presented as medians (25th–75th percentile). ^a^ Mann–Whitney *U* test.

**Table 6:** Proportions of long-chain polyunsaturated fatty acids in cord serum in relation to allergy diagnosis at 3 and 8 years of age.

Fatty Acid, % of Total Fatty Acids	Non-Allergic at 3 Years of Age (*n* = 37)	Allergic at 3 Years of Age (*n* = 10)	*p* ^a^	Non-Allergic at 8 Years of Age (*n* = 25)	Allergic at 8 Years of Age (*n* = 8)	*p* ^a^
20:5 n-3 (EPA)	0.30 (0.21–0.37)	0.20 (0.15–0.26)	**0.018**	0.31 (0.23–0.42)	0.21 (0.17–0.27)	**0.036**
22:5 n-3 (DPA)	0.25 (0.19–0.34)	0.22 (0.16–0.25)	0.286	0.22 (0.15–0.34)	0.22 (0.16–0.29)	0.984
22:6 n-3 (DHA)	4.1 (3.0–4.8)	3.7 (3.2–4.2)	0.310	4.1 (3.3–4.8)	4.0 (3.5–4.7)	0.918
20:4 n-6 (AA)	13 (11–14)	12 (10–14)	0.297	13 (11–14)	13 (12–15)	0.885
n-3 LCPUFA, sum	4.6 (3.7–5.6)	4.1 (3.6–4.8)	0.221	4.6 (3.9–5.6)	4.4 (3.8–5.2)	0.853
n-6 LCPUFA, sum	17 (15–18)	16 (14–17)	0.221	17 (16–19)	167(15–20)	0.885

Data are presented as medians (25th–75th percentile) of proportions of fatty acids (% of total fatty acids) in cord serum. ^a^ Mann–Whitney *U* test, significant differences are marked with bold. Abbreviations: AA, arachidonic acid; EPA, eicosapentaenoic acid; DPA, docosapentaenoic acid; DHA, docosahexaenoic acid; LCPUFA, long-chain polyunsaturated fatty acid. For proportions of all fatty acids in cord serum, see Supplementary Materials Table S2.

## Supplementary Materials

The following are available online at https://www.mdpi.com/2072-6643/12/10/3000/s1, Table S1: Monoclonal antibodies used to identify different B- and T-cell subsets. Table S2: Proportions of fatty acids in cord serum in relation to allergy diagnosis at 3 and 8 years of age.
