# Supplementary material for: Cord Blood Levels of EPA, a Marker of Fish Intake, Correlate with Infants’ T- and B-Lymphocyte Phenotypes and Risk for Allergic Disease
Source: Nutrients. 2020 Sep 30;12(10):3000. doi: 10.3390/nu12103000 (PMC7601506; doi:10.3390/nu12103000)
Supplement: Supplementary file 1 [file nutrients-12-03000-s001.pdf]

nutrients

MDPI

Table S1. Monoclonal antibodies used to identify different B- and T-cell subsets

Monoclonal antibody	Fluorochrome	Clone	Source
anti-CD5	APC	UCHT2	BD Bioscience,
anti-CD20	PerCP	L27	BD Bioscience
anti-CD45RA	FITC	L48	BD Bioscience
anti-CD45RO	PE	UCHL-1	BD Bioscience
anti-CCR4	PE	IG1	BD Bioscience
anti-CD25	APC	2A3	BD Bioscience
anti-FOXP3	PE	PCH101	eBioscience
anti-CD49d (α4)	FITC	44H6	BioRad (former AbD Serotec),
anti-β7-integrin	PE	FIB504	BD Bioscience
anti-CD4	PerCP	SK3	BD Bioscience

Abbreviations: APC, Allophycocyanin; PerCp, Peridinin Cholorophyll Protein Complex; FITC, fluorescein isothiocyanate; and PE, R-Phycoerythrin.

Table S2. Proportions of fatty acids in cord blood in relation to allergy diagnosis at 3 and 8 years of age.

Fatty acid	Non-Allergic at 3 Years of Age (*n* = 37)	Allergic at 3 Years of Age (*n* = 10)	*p* [a]	Non-Allergic at 8 Years of Age (*n* = 25)	Allergic at 8 Years of Age (*n* = 8)	*p* [a]
14:0	0.94 (0.71-1.1)	0.87 (0.73-1.1)	0.654	0.80 (0.68-1.1)	0.987 (0.66-1.1)	0.614
16:0	32 (31-34)	33 (31-34)	0.617	32 (31-33)	32 (31-34)	0.450
18:0	12 (11-12)	12 (11-12)	0.673	12 (11-13)	11 (9-12)	0.313
20:0	0.31 (0.26-0.38)	0.28 (0.27-0.35)	0.600	0.34 (0.29-0.39)	0.27 (0.23-0.39)	0.208
22:0	0.44 (0.34-0.56)	0.39 (0.34-0.51)	0.465	0.49 (0.38-0.60)	0.36 (0.24-0.60)	0.153
24:0	0.30 (0.22-0.43)	0.28 (0.23-0.30)	0.221	0.33 (0.26-0.46)	0.25 (0.17-0.38)	0.120
Saturated fatty acids, sum	*46 (44-47)*	*47 (45-48)*	*0.497*	*46 (44-47)*	*46 (43-47)*	*0.705*
16:1 n-7	4.2 (3.4-4.5)	3.8 (3.6-5.4)	0.274	3.7 (3.1-4.6)	4.0 (3.5-5.2)	0.401
18:1 n-7	3.6 (3.3-3.8)	3.6 (3.4-4.9)	0.434	3.6 (3.2-3.8)	3.6 (3.4-4.6)	0.334
18:1 n-9	20 (18-20)	20 (17-22)	0.808	20 (18-20)	20 (18-22)	0.769
Monounsaturated fatty acids, sum	*27 (25-29)*	*27 (25-33)*	*0.769*	*27 (25-29)*	*27 (25-32)*	*0.614*
18:2 n-6 (LA)	9.3 (8.6-10)	12 (10-14)	0.348	9.3 (8.6-10)	8.9 (8.2-10)	0.475
20:3 n-6	3.2 (2.8-3.7)	2.8 (2.3-3.3)	0.151	3.1 (2.8-3.9)	2.8 (2.2-3.3)	0.101
20:4 n-6 (AA)	13 (12-14)	12 (10-14)	0.297	13 (11-14)	13 (12-15)	0.867
22:4 n-6	0.33 (0.29-0.40)	0.31 (0.26-0.36)	0.513	0.33 (0.27-0.41)	0.35 (0.34-0.39)	0.450
22:5 n-6	0.38 (0.30-0.48)	0.45(0.30-0.58)	0.263	0.37 (0.29-0.49)	0.52(0.42-0.66)	0.026
n-6 PUFA, sum	*26 (25-28)*	*25 (22-27)*	*0.252*	*26 (25-28)*	*25 (24-29)*	*0.334*
n-6 LCPUFA, sum	*17 (16-18)*	*16 (14-17)*	*0.221*	*17 (16-19)*	*18 (15-20)*	*0.867*

18:3 n-3 (ALA)	0.12 (0.10-0.15)	0.10 (0.08-0.18)	0.636	0.12 (0.08-0.15)	0.11 (0.08-0.19)	1.000
20:5 n-3 (EPA)	0.30 (0.21-0.37)	0.20 (0.15-0.26)	0.018	0.31 (0.23-0.42)	0.21 (0.17-0.27)	0.036
22:5 n-3 (DPA)	0.25 (0.20-0.34)	0.22 (0.16-0.24)	0.286	0.22 (0.15-0.34)	0.22 (0.16-0.29)	0.966
22:6 n-3 (DHA)	4.1 (3.3-4.8)	3.7 (3.3-4.2)	0.310	4.1 (3.3-4.8)	4.0 (3.5-4.7)	0.900
n-3 PUFA, sum	4.7 (3.9-5.6)	4.2 (3.8-44.7)	0.211	4.7 (4.0-5.7)	4.5 (4.0-5.4)	0.834
n-3 LCPUFA, sum	4.6 (3.7-5.5)	4.1 (3.7-4.7)	0.221	4.6 (3.9-5.7)	4.4 (3.8-5.2)	0.834

Data are presented as medians (25th-75th percentile), [a]Mann-Whitney U test, Abbreviations: LA, linoleic acid; AA, arachidonic acid; ALA, alpha-linolenic acid; EPA, eicosapentaenoic acid; DPA, docosapentaenoic acid; DHA, docosahexaenoic acid; PUFA, polyunsaturated fatty acid, LCPUFA, long chain PUFA.
